# Geographic Variation in Active Perinatal Management and Outcomes for Extremely Preterm Infants in New South Wales: A Population‐Based Cohort Study

**DOI:** 10.1111/ajo.70196

**Published:** 2026-11-04

**Authors:** Nicholas Williams, Chenxi Li, Natasha Nassar, Adrienne Gordon

**Affiliations:** ^1^ RPA Newborn Care Royal Prince Alfred Hospital Sydney Australia; ^2^ Reproduction and Perinatal Centre, Faculty of Medicine and Health The University of Sydney Sydney Australia; ^3^ Implementation Science Academy Sydney Health Partners Sydney Australia; ^4^ Child Population and Translational Health Research, Children's Hospital at Westmead Clinical School, Faculty of Medicine and Health The University of Sydney Sydney Australia; ^5^ Leeder Centre for Health Policy, Economics and Data, Faculty of Medicine and Health The University of Sydney Sydney Australia; ^6^ Charles Perkins Centre The University of Sydney Sydney Australia

**Keywords:** delivery room, extremely premature infant, perinatal care, physicians' practice patterns, survival rate

## Abstract

**Background:**

Perinatal management of infants born at 22–25 weeks' gestation varies internationally and may influence survival and long‐term outcomes. In New South Wales (NSW), no contemporary statewide guideline exists.

**Aims:**

To evaluate rates of active delivery room management and associated survival and neurodevelopmental outcomes among infants born at 22–25 weeks' gestation in NSW.

**Materials and Methods:**

Population‐based cohort study of all live births at 22 + 0–25 + 6 weeks in NSW (2011–2019) using linked administrative and clinical datasets. Active management was defined as provision of respiratory support or higher in the delivery room. Primary outcomes were survival to 1 year and moderate or severe neurodevelopmental impairment (NDI) at 2–3 years.

**Results:**

Among 1285 live‐born infants, 69.0% received active management, increasing from 61.7% in 2011 to 74.5% in 2019 (*p* < 0.001). Rates varied across local health districts (54.9%–83.5%). One‐year survival was 44.9% overall (64.4% among actively managed infants) and increased with gestational age. Of 577 children eligible for follow‐up, 440 (76.3%) underwent assessment at 2–3 years, of whom 18.4% had moderate or severe NDI. Maternal transfer to a tertiary centre was associated with a lower adjusted hazard of death (HR 0.79, 95% CI 0.63–0.98) but not with NDI.

**Conclusions:**

Active management and survival increased over time; however, marked regional variation persists. Development of a statewide clinical practice guideline could support equitable, evidence‐based care for extremely preterm births.

## 1 Introduction

Extremely preterm birth at 22–25 weeks' gestation lies at the threshold of viability, where survival rises steeply with each additional week and decisions about active obstetric and neonatal care, including in utero transfer, are complex and time‐critical [1, 2, 3, 4]. Although gestational age strongly predicts outcomes, birth weight, sex, plurality, and antenatal corticosteroid exposure also influence prognosis [5, 6, 7]. Structured, prognosis‐based approaches have therefore been proposed to guide decision‐making and improve consistency in counselling and management [6]. At some periviable gestations, uncertainty regarding survival, the burdens of treatment, and long‐term outcomes mean that more than one management pathway may be ethically defensible. Contemporary frameworks consequently combine individualised prognostic assessment with shared decision‐making, allowing parental values and preferences to inform care within clinically reasonable limits [2, 8].

Internationally, wide variation in active perinatal management and outcomes has been documented. Recent United States data show ongoing between‐hospital differences in active treatment rates despite advances in perinatal care [9]. In the Neonatal Research Network, rates ranged ten‐fold across centres [10], and similar patterns were seen in the French EPIPAGE‐2 [11] and Swedish EXPRESS [12, 13] cohorts. Consistent proactive care was associated with higher survival without increased morbidity.

Within Australia, the most recent NSW consensus guideline for births below 26 weeks was published 20 years ago in 2006, based on data from 1998 to 2000 [14]. Since then, survival and long‐term neurodevelopmental outcomes for extremely preterm infants have dramatically improved [15, 16, 17, 18, 19], and clinician and parent attitudes have shifted towards more proactive care [20]. In the absence of updated statewide guidance, several tertiary centres and local health districts (LHDs) have developed independent protocols, resulting in divergent practice. A recent national study confirmed that care of infants at 22–26 weeks' gestation does not consistently follow expert recommendations [21]. Evidence from Victoria shows that implementation of the 2020 Safer Care Victoria Extreme Prematurity Guideline increased active‐care rates and improved survival [2, 22, 23]. These findings underscore how inconsistent policy frameworks can perpetuate inequity in survival‐focused care.

This variation contradicts Australia's commitment to equitable access and consistent quality under the National Health Reform Agreement [24] and the safety‐and‐quality standards of the Australian Commission on Safety and Quality in Health Care [25]. Persistent disparities in specialist access for people living outside major cities [26, 27] indicate that differences in perinatal management reflect not only clinical factors but also the performance of the broader health system.

We therefore aimed to quantify regional variation in active perinatal management for infants at 22–25 weeks' gestation in NSW and to examine associations with one‐year survival and neurodevelopmental outcomes at 2–3 years, interpreting these findings within current Australian policy frameworks for equitable, standardised perinatal care.

## 2 Materials and Methods

### 2.1 Study Design and Data Sources

We conducted a population‐based, data‐linkage cohort study of infants born at 22 + 0–25 + 6 weeks' gestation in New South Wales (NSW) between 1 January 2011 and 31 December 2019. Data were obtained from routinely collected statewide datasets: the NSW Perinatal Data Collection (PDC), Admitted Patient Data Collection (APDC), Registry of Births, Deaths and Marriages (RBDM), Neonatal Intensive and Special Care Units (NICUS) Registry, and the Australian Cerebral Palsy Register (CPR).

The PDC captures all births at ≥ 20 weeks' gestation or with a birth weight of ≥ 400 g, including maternal demographics, pregnancy care, and birth outcomes. Maternal conditions, transfers, neonatal morbidity, and congenital anomalies were extracted from the APDC. The NICUS registry provided data on neonatal management and follow‐up outcomes, and cerebral palsy diagnoses were verified through both NICUS and the CPR. Mortality data were ascertained from RBDM registrations.

The NSW Centre for Health Record Linkage (CHeReL) performed deterministic linkage using de‐identified, project‐specific person numbers. Ethics approval was granted by the NSW Population and Health Services Research Ethics Committee (2019/ETH11532). The study adhered to the Strengthening the Reporting of Observational Studies in Epidemiology (STROBE) guidelines.

### 2.2 Setting and Participants

We included all births at 22 + 0–25 + 6 weeks' gestation in NSW during the study period. NSW is Australia's most populous state, accounting for almost one‐third of national births, with around 95 000 annually and 23% occurring outside major cities [26].

Public maternity and neonatal services in NSW are administered through 15 Local Health Districts (LHDs), six metropolitan and nine regional or rural [28]. A tertiary maternity hospital was defined as one providing antenatal, intrapartum, and postnatal care for infants < 32 weeks' gestation [29].

We identified live births from the PDC and excluded infants with lethal congenital anomalies, terminations of pregnancy, or implausible birthweights (> 3000 g).

### 2.3 Study Outcomes

Primary outcomes were rates of active delivery‐room management, in utero maternal transfer, one‐year survival, and moderate or severe neurodevelopmental impairment (NDI) at 2–3 years. Active delivery‐room management was defined as provision of continuous positive airway pressure (CPAP) or higher‐level intervention (intermittent positive‐pressure ventilation, intubation, chest compressions, resuscitation drugs or fluids, or mechanical ventilation). Infants receiving only oxygen or suctioning were classified as non‐active, as these may occur during palliative care. Maternal transfer was analysed separately because it influences both survival and service utilisation.

NDI was obtained from the NICUS Registry data, incorporating Bayley‐III developmental scores, diagnoses of cerebral palsy, and sensory impairment. Cerebral palsy was classified using the Gross Motor Function Classification System (GMFCS) [30]. Bayley‐III cognitive, motor, and language scores were categorised as mild (70–84), moderate (55–69), or severe (< 55) delay. Blindness (< 6/60 in the better eye) and deafness requiring hearing aids or cochlear implants were also included. Functional impairment was categorised as mild (GMFCS I or mild Bayley delay), moderate (GMFCS II–III, deafness, or moderate delay), or severe (GMFCS IV–V, blindness, or severe delay).

### 2.4 Maternal and Infant Factors

Maternal characteristics included age, country of birth, parity, smoking, hypertension, diabetes, antepartum haemorrhage, presentation and birth hospital, and residence postcode. Postcode was used to determine geographic remoteness and socioeconomic status based on the Socio‐Economic Indexes for Areas (SEIFA) [31].

Infant factors included birthweight, gestational age, sex, plurality, antenatal corticosteroid exposure, and Apgar scores at 1 and 5 min. Small‐for‐gestational‐age (SGA) was defined as < 10th percentile.

### 2.5 Statistical Analysis

Characteristics of infants receiving active versus non‐active management were compared using chi‐square and *t*‐tests. Active management rates were summarised by gestational age, year of birth, and LHD. Trends were tested using the Cochran‐Armitage test.

Multivariable logistic regression was used to compare active management between LHDs, adjusted for maternal age, socioeconomic status, and country of birth. Kaplan–Meier survival curves and Cox regression models estimated one‐year survival; logistic regression identified factors associated with NDI. Analyses were performed using SAS v9.4 and R v4.1.3. Statistical significance was defined as *p* < 0.05 or 95% confidence intervals excluding one.

## 3 Results

### 3.1 Cohort Characteristics and Maternal Transfers

Between 2011 and 2019, 2841 infants were born at 22–25 weeks' gestation in NSW. Of these, 1360 were stillborn and 196 were excluded (lethal anomalies, terminations, or implausible birth weights), leaving 1285 live‐born infants (Figure 1). The number increased with gestational age from 197 at 22 weeks to 457 at 25 weeks.

**FIGURE 1:**
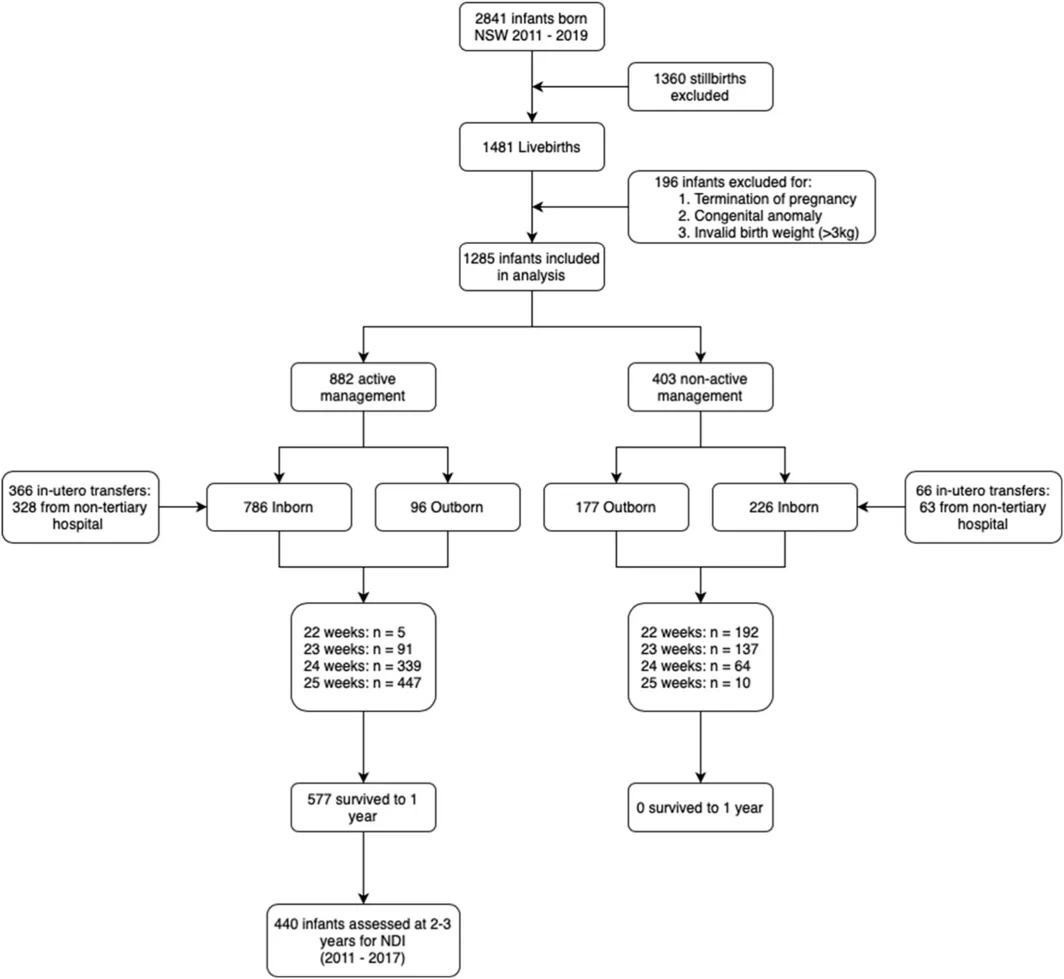
Flow diagram of infants included in the study.

Active management rates varied by maternal and infant characteristics (Table 1). Only 32 of 62 infants (51.6%) born to mothers ≤ 19 years received active management, compared with 69.5% among older mothers. Actively managed infants were of higher gestational age and birthweight, less likely to be SGA, and had higher Apgar scores. Male infants were more likely than females to receive active management (56.7% vs. 49.1%; *p* = 0.014).

**TABLE 1:** Perinatal characteristics of live births with gestational age between 22 and 25 completed weeks in NSW from 2011 to 2019.

Maternal and infant characteristics	Non‐active management (*n* = 403)	Active management (*n* = 882)	*p*
**Maternal characteristics**			
Age *n* (%)			
≤ 19 years	30 (7.4)	32 (3.6)	0.012
20–34 years	275 (68.2)	622 (70.5)	
≥ 35 years	98 (24.3)	228 (25.9)	
Mother born in Australia *n* (%)	256 (63.5)	552 (62.6)	0.535
Maternal residence			
Metropolitan	320 (79.4)	703 (79.7)	0.976
Non‐metropolitan	75 (18.6)	163 (18.5)	
Socioeconomic disadvantage (SEIFA) *n* (%)			
Q1‐ most disadvantaged	92 (22.8)	250 (28.3)	0.103
Q2	64 (15.9)	135 (15.3)	
Q3	80 (19.9)	169 (19.2)	
Q4	96 (23.8)	156 (17.7)	
Q5‐ least disadvantaged	59 (14.6)	141 (16.0)	
Maternal smoking *n* (%)	54 (13.4)	149 (16.9)	0.131
Primipara *n* (%)	221 (54.8)	490 (55.6)	0.710
IVF Pregnancy *n* (%)	32 (7.9)	49 (5.6)	0.131
Received antenatal care *n* (%)	383 (95.0)	834 (94.6)	0.290
Maternal hypertension *n* (%)	25 (6.2)	94 (10.7)	0.014
Gestational diabetes *n* (%)	34 (8.4)	99 (11.2)	0.155
Antenatal steroids *n* (%)^a^			
None	NR	58 (8.0)	
< 24 h	NR	246 (33.7)	
> 7 days	NR	61 (8.4)	
Complete	NR	364 (49.9)	
Antenatal magnesium sulphate exposure *n* (%)^a^	NR		
**Birth characteristics**			
Antepartum haemorrhage *n* (%)	89 (22.1)	177 (20.1)	0.451
Any maternal transfer from non‐tertiary to tertiary maternity hospital	63 (15.6)	328 (37.2)	< 0.001
Onset of labour *n* (%)			
No Labour	13 (3.2)	215 (24.4)	< 0.001
Induced	52 (12.9)	23 (2.6)	
Spontaneous	338 (83.9)	644 (73.0)	
Birth by C‐section *n* (%)	15 (3.7)	362 (41.0)	< 0.001
Born in tertiary maternity hospital *n* (%)	226 (56.1)	786 (89.1)	< 0.001
**Infant characteristics**			
Singleton *n* (%)	311 (77.2)	664 (75.3)	0.507
Male sex *n* (%)	198 (49.1)	500 (56.7)	0.014
Birth Weight *n* (%)			
< 400 g	22 (5.5)	< 5	< 0.001
400–599 g	260 (64.5)	156 (17.7)	
600–799 g	109 (27.0)	475 (53.9)	
> 800 g	6 (1.5)	247 (28.0)	
Small for gestational age (≤ 10th centile) *n* (%)	53 (13.2)	77 (8.7)	< 0.001
Gestation *n* (%)			
22 weeks	192 (47.6)	5 (0.6)	< 0.001
23 weeks	137 (34.0)	91 (10.3)	
24 weeks	64 (15.9)	339 (38.4)	
25 weeks	10 (2.5)	447 (50.7)	
Apgar scores *n* (%)			
1 min ≤ 3	327 (81.1)	331 (37.5)	< 0.001
5 min ≤ 3	358 (88.8)	107 (12.1)	< 0.001

Abbreviations: NR, no result; SEIFA, socio‐economic index for areas.

^a^ Results available only for infants admitted to NICU [*n* = 729].

There were 442 in utero maternal transfers, mostly from non‐tertiary to tertiary hospitals (*n* = 391; 88.5%). Transfers between tertiary hospitals (*n* = 41) and between non‐tertiary facilities (*n* = 10) were less frequent. Among those transferred from non‐tertiary to tertiary centres, 63 infants (16.1%) subsequently received palliative care. Active management was more common among infants whose mothers had been transferred to a tertiary facility (37.2% vs. 15.6%; *p* < 0.001).

### 3.2 Active Management

The proportion of infants receiving active management increased with gestational age: 2.5% at 22 weeks, 39.9% at 23 weeks, 84.1% at 24 weeks, and 97.8% at 25 weeks. Across the decade, active management rose from 61.7% in 2011 to 74.5% in 2019 (*p* < 0.001; Figure S1).

Significant variation was observed across LHDs, with active management rates ranging from 0% to 84% (Table 2; Figure S2). Among LHDs containing tertiary maternity hospitals, rates ranged from 60% to 84%. Adjusted odds ratios for active management across districts ranged from 0.29 to 1.02 relative to the LHD with the highest rate.

**TABLE 2:** Active management rates by Local Health District (LHD) and odds of receiving active management.

Local health district	Number of births	Active management *n* (%)	Crude odds ratio (95% CI)	Adjusted odds ratio^a^ (95% CI)
Northern Sydney	133	111 (83.5)		Reference
Sydney	134	81 (60.4)	0.3 (0.17–0.54)	0.29 (0.16–0.53)
South Western Sydney	222	160 (72.1)	0.51 (0.3–0.88)	0.48 (0.27–0.84)
South Eastern Sydney	169	130 (76.9)	0.66 (0.37–1.18)	0.68 (0.37–1.23)
Western Sydney	187	123 (65.8)	0.38 (0.22–0.66)	0.37 (0.21–0.66)
Nepean Blue Mountains	120	100 (83.3)	0.99 (0.51–1.92)	1.04 (0.51–2.12)
Hunter New England	188	126 (67.0)	0.4 (0.23–0.7)	0.34 (0.18–0.62)
Other LHD^b^	102	47 (46.1)	0.17 (0.09–0.31)	0.16 (0.08–0.31)

^a^ Regression analysis accounting for maternal age, maternal country of birth and socio‐economic status (SEIFA).

^b^ LHDs without a tertiary maternity unit & NICU have been grouped under “Other LHD”.

### 3.3 Neonatal Survival

Overall, 44.9% of live‐born infants survived to 1 year. Among those receiving active management, survival was 64.4%. Survival increased with gestational age, from 0% at 22 weeks, 19.2% at 23 weeks, 47.6% at 24 weeks, and 73.9% at 25 weeks. For actively managed infants, respective survival rates were 0%, 48.2%, 56.7%, and 75.5% (Figure 2).

**FIGURE 2:**
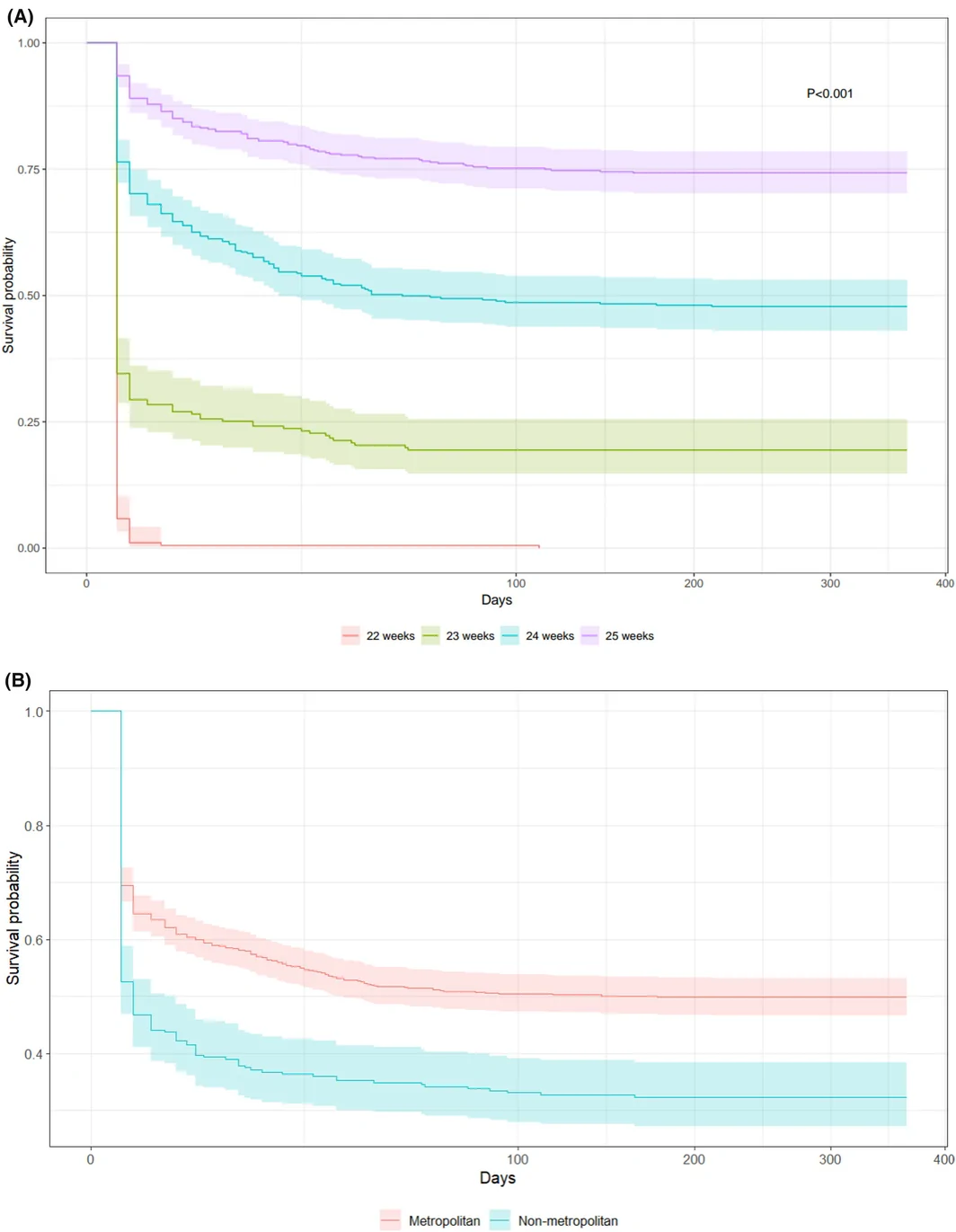
One‐year survival probability curves by gestation age (A) and geographical location of birth (B) for infants who received active management in the delivery room.

One‐year survival of live‐born infants improved from 35.9% in 2011 to 47.0% in 2019, coinciding with increased active management.

In multivariable Cox regression, increasing gestational age was associated with lower mortality risk (hazard ratio [HR] per week 0.37; 95% CI 0.33–0.41). Maternal transfer to a tertiary centre was also associated with reduced mortality (HR 0.79; 95% CI 0.63–0.98). SGA (HR 1.69; 95% CI 1.27–2.24) and residence in a non‐metropolitan LHD (HR 1.51; 95% CI 1.14–2.00) were associated with increased mortality.

### 3.4 Neurodevelopmental Outcomes

Among 577 infants born 2011–2017 who survived to 1 year, neurodevelopmental data were available for 440 (76.3%) at 2–3 years. Of the infants assessed, 67.8% had no or mild impairment and 7.3% had a diagnosis of cerebral palsy (Table S1). Rates of moderate–severe impairment did not vary significantly by gestational age (*p* > 0.05).

Factors associated with increased odds of moderate–severe impairment included maternal country of birth outside Australia (odds ratio 1.83; 95% CI 1.11–3.00), residence in the most socioeconomically disadvantaged areas (OR 1.84; 95% CI 1.12–3.03), male sex (OR 2.36; 95% CI 1.40–3.99), and SGA (OR 2.18; 95% CI 1.00–4.77). After adjustment, maternal transfer was not associated with impairment (adjusted OR 1.35; 95% CI 0.79–2.30).

## 4 Discussion

In this large, population‐based cohort study of infants born at 22–25 weeks' gestation in New South Wales (NSW), we identified substantial variation in the provision of active perinatal management and associated outcomes across the state. Infants born in non‐metropolitan hospitals were less likely to receive active management and had higher mortality, even after adjustment for clinical and maternal factors. However, variation was not confined to rural areas: marked differences also occurred among metropolitan LHDs, including those containing tertiary maternity hospitals. These findings suggest that survival‐focused care at the threshold of viability is not applied consistently across NSW.

Internationally, variation in active perinatal management has long been recognised as a determinant of outcomes. In the United States, between‐hospital differences in active treatment persisted over the past decade, with large datasets showing ongoing practice variation despite advances in neonatal care [9, 10]. Similarly, in the French EPIPAGE‐2 cohort, inter‐unit variation in neonatal care explained differences in mortality and two‐year neurodevelopmental outcomes among infants born at 27–31 weeks [11]. Conversely, the Swedish EXPRESS cohorts demonstrated that consistent, proactive management improves one‐year survival without increasing morbidity [12, 13]. Our results mirror these international experiences and add to Australian data showing that national practice continues to diverge from expert recommendations [21]. Together, these findings indicate that inconsistent application of active perinatal care may contribute to variation in outcomes.

A likely explanation for the variation in the provision of active management in NSW is the absence of a contemporary, statewide perinatal guideline or framework for births at 22–25 weeks. In its absence, individual LHDs and tertiary centres have developed local protocols, resulting in inconsistent thresholds for initiating active management. This decentralised approach contrasts with Victoria, where the Safer Care Victoria Extreme Prematurity Guideline provides unified direction across maternity and neonatal services [2]. Implementation of this guideline was associated with increased rates of active management [23]. Similar approaches internationally, such as in Sweden, Japan, and other high‐income countries, have shown that standardised policies improve both consistency of care and outcomes [11, 12, 13, 19]. Establishing an equivalent state or national framework in Australia could reduce unwarranted variation and align decision‐making with contemporary evidence.

These findings also raise broader questions about equity and health‐system governance. Under the National Health Reform Agreement [24], states and territories are responsible for ensuring equitable access to health services, however, our data suggest that equitable access to survival‐focused care may not yet be consistently achieved for the most vulnerable infants. The Australian Commission on Safety and Quality in Health Care identifies equity and consistency as core dimensions of quality [25], while national data continue to highlight gaps in specialist access for people living outside major cities [26, 27]. Variation in active perinatal management may reflect broader health‐system fragmentation rather than solely clinical factors.

From a clinical standpoint, the strong association between active management and survival underscores the importance of coordinated obstetric and neonatal decision‐making. At some periviable gestations, prognostic uncertainty means that both survival‐focused and palliative care may be ethically defensible. Variation in management is therefore not inherently inappropriate when it reflects individual prognosis and informed parental values [8]. However, geographic variation is concerning if families receive different treatment options, prognostic information, counselling, or access to tertiary expertise according to where they present. Contemporary guidelines can define the clinically reasonable range of options while supporting consistent, individualised shared decision‐making [2].

Strengths of this study include its population‐based design, comprehensive linkage across multiple state datasets, and inclusion of both survival and long‐term neurodevelopmental outcomes. Limitations include its retrospective nature, use of delivery‐room management as a proxy for antenatal intent, and incomplete follow‐up for some survivors. Hospital‐level data on workforce, staffing models, or local guideline differences were unavailable and may partly explain regional variation. Nevertheless, the size and coverage of this cohort minimise selection bias and provide robust, representative insight into practice variation across NSW.

Taken together, these findings suggest that active perinatal management for extremely preterm infants varies substantially across NSW, including among LHDs with tertiary maternity services. Given the strong association between active management and survival, this variation may contribute to inequities in outcomes. The absence of a contemporary statewide perinatal guideline and reliance on locally developed protocols may partly explain these differences. Development of coordinated, evidence‐informed guidance could support more consistent decision‐making and promote greater equity in access to perinatal care.

## Funding

The authors have nothing to report.

## Conflicts of Interest

The authors declare no conflicts of interest.

## Supporting information


**Figure S1:** Delivery room active management and survival for all live births by year for infants born 22–25 weeks' gestation.
**Figure S2:** Rates of active management by Local Health District.
**Table S1:** Neurodevelopmental impairment at two to 3 years of age follow‐up by gestational age at birth.

## Data Availability

The data that support the findings of this study are available from the NSW Ministry of Health, NICUS Data Registry and the Australian Cerebral Palsy Register. Restrictions apply to the availability of these data, which were used under licence for this study. Data may be made available with the permission of the relevant data custodians and subject to appropriate ethical and governance approvals.
