# Supplementary material for: Geographic Variation in Active Perinatal Management and Outcomes for Extremely Preterm Infants in New South Wales: A Population‐Based Cohort Study
Source: Aust N Z J Obstet Gynaecol. 2026 Nov 4;66(6):e70196. doi: 10.1111/ajo.70196 (PMC13732407; doi:10.1111/ajo.70196)
Supplement: Supplementary file 1 [file AJO-66-0-s001.docx]

**Supplementary Material**

 *Supplementary Figure 1: Delivery room active management and survival for all live births by year for infants born 22 – 25 weeks’ gestation*

*Supplementary Figure 2: Rates of active management by Local Health District*

| **Neurodevelopmental Impairment** | **23 weeks**  **(n=30)** | **24 weeks**  **(n=148)** | | | **25 weeks**  **(n=262)** | | **Overall**  **(n=440)** |  |
| --- | --- | --- | --- | --- | --- | --- | --- | --- |
| Cerebral Palsy n (%) | <5 | 13 (8.8) | | | 17 (6.5) | | 32 (7.3) |  |
| Bayley Assessment Moderate-Severe Neurodevelopmental Delay n (%) | | | | | | | | |
| Motor | <5 | 10 (6.8) | | | 22 (8.4) | | 34 (7.7) |  |
| Language | 5 (16.7) | 24 (16.3) | | | 27 (10.3) | | 56 (12.7) |  |
| Cognitive | <5 | 10 (6.8) | | | 12 (4.6) | | 24 (5.5) |  |
| Functional Impairment level n (%) | | | | | | | |  |
| Unknown | <5 | | 19 (12.8) | 38 (14.5) | | 61 (13.9) | | |
| None | 10 (33.3) | | 62 (41.9) | 117 (44.7) | | 189 (43.0) | | |
| Mild | 10 (33.3) | | 36 (24.3) | 63 (24.0) | | 109 (24.8) | | |
| Moderate | <5 | | 22 (14.9) | 34 (13.0) | | 58 (13.2) | | |
| Severe | <5 | | 9 (6.1) | 10 (3.8) | | 23 (5.2) | | |

*Supplementary Table 1: Neurodevelopmental impairment at two to three years of age follow-up by gestational age at birth*
